# First *in vivo* Evidence That Glutathione-S-Transferase Operates in Photo-Oxidative Stress in Cyanobacteria

**DOI:** 10.3389/fmicb.2019.01899

**Published:** 2019-08-13

**Authors:** Xavier Kammerscheit, Franck Chauvat, Corinne Cassier-Chauvat

**Affiliations:** Institute for Integrative Biology of the Cell (I2BC), CEA, CNRS, Univ Paris-Sud, Université Paris-Saclay, Gif-sur-Yvette, France

**Keywords:** *Synechocystis* PCC 6803, light tolerance, methylene blue, menadione, hydrogen peroxide, catalase-peroxidase, glutathione

## Abstract

Although glutathione (GSH) and GSH-dependent enzymes, such as glutathione transferases (GSTs), are thought to have been developed by cyanobacteria to cope with the reactive oxygen species (ROS) that they massively produced by their active photosynthesis, there had been no *in vivo* analysis of the role of GSTs in cyanobacteria so far. Consequently, we have analyzed two of the six GSTs of the model cyanobacterium *Synechocystis* PCC 6803, namely Sll1545 (to extend its *in vitro* study) and Slr0236 (because it is the best homolog to Sll1545). We report that Sll1545 is essential to cell growth in standard photo-autotrophic conditions, whereas Slr0236 is dispensable. Furthermore, both Sll1545 and Slr0236 operate in the protection against stresses triggered by high light, H_2_O_2_, menadione and methylene blue. The absence of Slr0236 and the depletion of Sll1545 decrease the tolerance to methylene blue in a cumulative way. Similarly, the combined absence of Slr0236 and depletion of Sll1545 decrease the resistance to high light. Attesting their sensitivity to high-light or methylene blue, these Δ*slr0236-sll1545* cells transiently accumulate ROS, and then reduced and oxidized glutathione in that order. In contrast, the absence of Slr0236 and the depletion of Sll1545 increase the tolerance to menadione in a cumulative way. This increased menadione resistance is due, at least in part, to the higher level of catalase and/or peroxidase activity of these mutants. Similarly, the increased H_2_O_2_ resistance of the Δ*slr0236-sll1545* cells is due, at least in part, to its higher level of peroxidase activity.

## Introduction

Glutathione, the highly abundant (1–10 mM) tripeptide L-glutamyl-L-cysteinyl-L-glycine (Lu, 2013), plays a crucial role in cell resistance to oxidative and metabolic stresses in most organisms. Glutathione occurs under two forms (Zhang and Forman, 2012; Lu, 2013). The reduced (major) form (GSH) maintains the intracellular compartment in a reduced state and supplies electrons to various enzymes, such as glutaredoxins, glutathione peroxidases and glutathione-S-transferases (GSTs) that detoxify reactive oxygen species (ROS), xenobiotics and/or heavy metals (Yadav, 2010; Noctor et al., 2012). The resulting oxidized form of glutathione, the dimeric disulfide form (GSSG), can be reduced back to GSH by various factors, such as the NADPH-using enzyme glutathione reductase (GR) that occurs in many but not all organisms (Fahey, 2013). For example, the presently studied model cyanobacterium *Synechocystis* PCC 6803 has no GR (Marteyn et al., 2009; Naraimsamy et al., 2013). ROS can also function in signaling but it is important to note that the changes in ROS required for signaling do not cause significant changes in intracellular ratio of GSH to GSSG signaling (Schieber and Chandel, 2014).

The superfamily of GSTs (EC 2.5.1.18) catalyzes the nucleophilic attack by the reduced (thiol) sulfur atom (SH) of the cysteinyl residue of GSH on the electrophilic center of diverse hydrophobic compounds (R-X). This generates water-soluble glutathione conjugates linked by a thioether bond (GS-R) that can then be degraded, or excreted out of the cell (Deponte, 2013). Physiologically, this transferase activity operates in the detoxification of endogenous ROS (Noctor et al., 2012) and peroxides compounds, as well as xenobiotics, heavy metals (Yadav, 2010; Noctor et al., 2012) and phytotoxics (for review, see Nianiou-Obeidat et al., 2017).

Glutathione-S-transferases can also display a GSH-dependent thiol-transferase activity that generates disulfide bridges, which can link the thiols of the cysteinyl residue of two molecules of GSH, yielding GSSG (Deponte, 2013), or the cysteinyl thiols of one molecule of GSH and of one cysteine residue of a protein (a phenomenon termed glutathionylation) (Manevich et al., 2004; Carvalho et al., 2016). The reduction of these disulfide bridges (called deglutathionylation) can be catalyzed by various enzymes (glutaredoxins, thioredoxins) including GSTs (for a recent review, see Sylvestre-Gonon et al., 2019). In photosynthetic organisms, deglutathionylation was found to operate in redox regulation, protection and recovery of oxidized enzymes (Moons, 2005; Marteyn et al., 2013; Nianiou-Obeidat et al., 2017).

Glutathione-S-transferases can also have GSH-dependent peroxidase (GPOX) and dehydroascorbate reductase (GDHAR) activities (Moons, 2005). For example, in plants a tau-class GST operates in the transcriptional activation of flavonoid biosynthesis genes involved in cell defenses (Moons, 2005).

In addition, GSTs can also have a non-catalytic “ligandin” activity that binds non-substrate ligands (Oakley, 2011). In plants, this activity is involved in the cellular distribution of phytohormones and anthocyanin (Nianiou-Obeidat et al., 2017).

Glutathione-S-transferases are commonly divided in three different families: (i) cytosolic GSTs, (ii) mitochondrial GSTs, and (iii) microsomal (membranous) GSTs designated as MAPEGs (membrane-associated protein involved in ecosanoïd and glutathione metabolism) (Oakley, 2011). Cytosolic GSTs, which constitute the largest GST family, can be divided in many classes according to their protein sequence and structure (Oakley, 2011), the nature of their xenobiotic substrates and their antibody cross reactivity (Blanchette et al., 2007). Members of a same cytosolic GSTs class possess greater than 40% amino-acid (aa) sequence identity, whereas GSTs belonging to different classes share less than 25% of aa identity (Oakley, 2011). The currently recognized classes of cytosolic GSTs are: alpha, beta, delta, epsilon, zeta, theta, lambda (Lallement et al., 2014), mu, nu, pi, sigma, tau, phi, and omega (for review, see Oakley, 2011). More recently, others GST classes were described in bacteria: nu, zeta, and eta (for review, see Shehu et al., 2018) and in cyanobacteria: chi (Wiktelius and Stenberg, 2007; Pandey et al., 2015) and rho (Pandey et al., 2017). The mitochondrial GSTs share an evolutionary pattern with the cytosolic GSTs but differ from them by their structure and their subcellular localization (Morel and Aninat, 2011). The MAPEG GSTs operate in the detoxification of lipid peroxidation (Johansson et al., 2010), or the biosynthesis of hormone (leukotrienes, prostaglandins, and steroids) (Ago et al., 2007; Oakley, 2011; Sjögren et al., 2013).

Very little is known about GSTs in cyanobacteria, even though these prokaryotes are regarded as the inventor of the oxygen-evolving photosynthesis (William Schopf, 2011), as well as the GSH and the GSH-dependent GST enzymes to protect themselves against the ROS that they massively produce by their active photosynthesis (William Schopf, 2011). ROS can also operate in signaling (see above). Furthermore, cyanobacteria produce a large of the biomass and oxygen for the food chain, have a high interest for basic research (Cassier-Chauvat and Chauvat, 2018). They are widely used to decipher the molecular processes of photosynthesis (they are viewed, as the ancestors of the plant chloroplast Archibald, 2009) and the related carbon fixation process (Montgomery et al., 2016), as well as nitrogen fixation and cell communication (Herrero et al., 2016), cell differentiation (Magnuson, 2019) and division. Moreover, cyanobacteria are increasingly studied and engineered for the future photosynthetic (carbon-neutral) production of high-value chemicals (Knoot et al., 2018). The realization of this important goal requires a better understanding of the cyanobacterial tolerance to stresses (Cassier-Chauvat et al., 2016), again highlighting the interest of studying the cyanobacterial GST.

The chi and rho classes of cytosolic GSTs were first identified by *in silico* and/or *in vitro* analyses of GST genes from the unicellular cyanobacteria *Synechococcus elongatus* PCC 6301 and *Thermosynechococcus elongatus* BP-1 for chi class (Wiktelius and Stenberg, 2007), and *Synechocystis* PCC 6803 for chi (Pandey et al., 2015) and rho classes (Pandey et al., 2017). The cyanobacterial chi GSTs produced in *Escherichia coli* were found to efficiently catalyze the conjugation of GSH to the plant defense isothiocyanate compounds (Wiktelius and Stenberg, 2007). The *Synechocystis* PCC 6803 rho GST produced in *E. coli* was shown to have a strong dichloro-acetate dehalogenase activity and a glutathione-dependent peroxidase activity with a high preference for cumene hydroxyperoxide (Pandey et al., 2017).

In the frame of our long-term interest in stress responses in cyanobacteria (for a review, see Cassier-Chauvat and Chauvat, 2014) we carried out the first *in vivo* analysis of cyanobacterial GSTs. For this purpose, we used the widely studied model *Synechocystis* PCC 6803 (hereafter *Synechocystis*) that possesses a small genome (about 4.0 Mb) comprising six chromosomal GST genes (designated as *sll0067*, *sll1147*, *sll1545*, *sll1902*, *slr0236*, and *slr0605* in cyanobase)^1^. We focused our attention on *sll1545* and *slr0236* because Sll1545 was shown *in vitro* to possess a genuine GST activity (Pandey et al., 2017) and Slr0236 because we found that it shares the greatest sequence identity with Sll1545 (about 32%). We show that Sll1545 is essential to the growth of *Synechocystis* in standard photo-autotrophic conditions, whereas Slr0236 is dispensable. Both Sll1545 and Slr0236 operate in the tolerance to stresses triggered by high light, H_2_O_2_, menadione and methylene blue. We also report on the impact of Sll1545 and Slr0236 on the kinetics of appearance and elimination of ROS, and the subsequent oxidation and re-reduction of glutathione used for their detoxification.

## Results

### The *sll1545* GST Gene Is Essential for the Viability of *Synechocystis* Whereas *slr0236* Is Dispensable for the Photoautotrophic Growth

**FIGURE 1:**
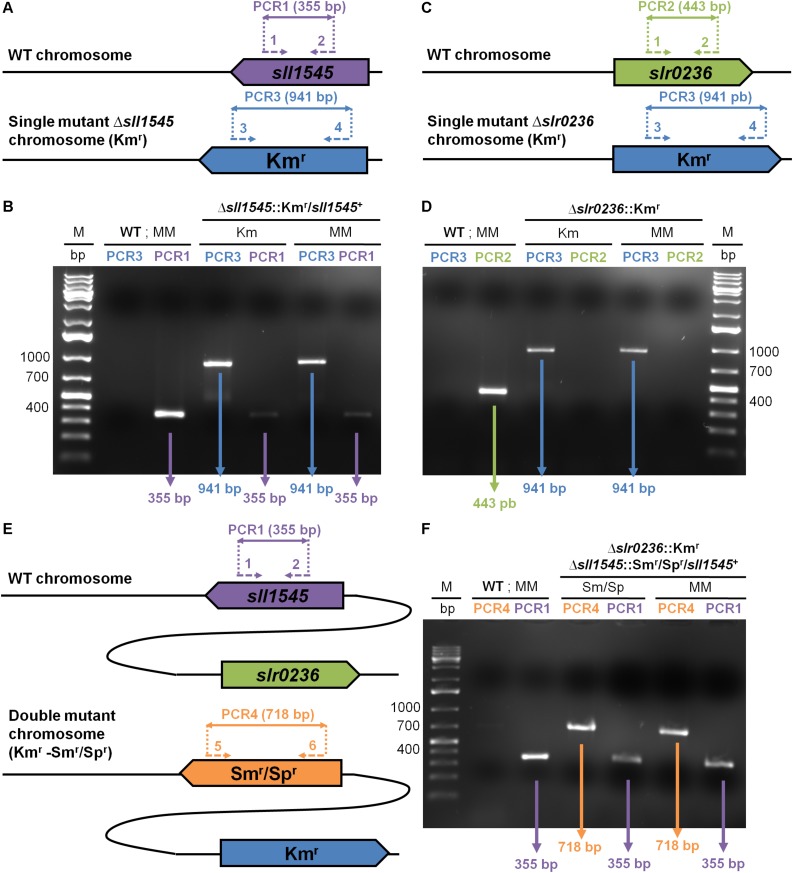
PCR analysis of the studied chromosome loci in *Synechocystis* WT strain and the mutants constructed in this study, which were grown in absence (MM) or presence of the selective antibiotics (Km or Sm/Sp). **(A,C,E)** The genes are represented by colored arrows (*sll1545*: purple; *slr0236*: green; Km^*r*^: blue, and Sm^*r*^/Sp^*r*^: orange) which point into the direction of their transcription. The same color code is used to represent the PCR primers (dotted arrows see Supplementary Table S2 for their sequence) and the corresponding PCR products (double arrows) typical of the presence of the WT, Km^*r*^ or Sm^*r*^/Sp^*r*^ chromosome copies. **(B,D,F)** Typical UV-light images of the agarose gels showing the PCR products corresponding to the genes *sll1545* (PCR1: purple), *slr0236* (PCR2: green), Km^*r*^ (PCR3: blue) and Sm^*r*^/Sp^*r*^ (PCR4: orange). M indicates the GeneRuler 1 kb Plus DNA Ladder (Thermo Scientific). Note that (1) WT cells harbors only WT chromosomes; (2) the Δ*slr0236*:Km^*r*^ mutant possesses only Δ*slr0236*:Km^*r*^ chromosomes; (3) the Δ*sll1545*:Km^*r*^/*sll1545*^+^ mutant has both WT (*sll1545*^+^) and mutant (Δ*sll1545*:Km^*r*^) copies of the chromosomes. The mutant Δ*slr0236*:Km^*r*^−−Δ*sll1545*:Sm^*r*^/Sp^*r*^-*sll1545*^+^ harbors only Δ*slr0236*:Km^*r*^ chromosome copies some of which possess the *sll1545*^+^ WT allele whereas the other possess the Δ*sll1545*:Sm^*r*^/Sp^*r*^ mutant allele.

To analyze *in vivo* the role of Sll1545 and Slr0236, the two best homologous GSTs of *Synechocystis* (about 32% of amino-acids sequence identity, data not shown), we constructed the deletion mutants of the *sll1545* and *slr0236* genes, using the standard procedure. Practically, the full protein-coding sequences of *sll1545* and *slr0236* were independently replaced by a transcription-terminator-less antibiotic-resistance gene Km^*r*^ or Sm^*r*^/Sp^*r*^ for selection, while preserving 300 bp of the *sll1545* and *slr0236* flanking DNA regions for homologous recombination mediating targeted gene replacement upon transformation to *Synechocystis* (Labarre et al., 1989). The resulting deletion cassettes cloned in an *E. coli* plasmid (Supplementary Table S1) were independently introduced in *Synechocystis* by transformation (Labarre et al., 1989). In each case a few (antibiotic resistant) transformants were selected and analyzed by PCR with specific oligonucleotide primers (Supplementary Table S2 and Figures 1A,C,E) to verify that the Km^*r*^ or Sm^*r*^/Sp^*r*^ marker genes had properly replaced *sll1545* or *slr0236* in the polyploïd *Synechocystis* chromosome (it occurs, at about ten copies per cell Labarre et al., 1989). Then, we assayed whether the segregation of WT and mutant (Km^*r*^ or Sm^*r*^/Sp^*r*^) chromosome copies was complete (the gene is dispensable to cell growth) or not (the gene is essential to cell viability).

All Δ*slr0236*:Km^*r*^ transformants possessed only Δ*slr0236*:Km^*r*^ chromosomes [Figure 1D, see the presence of a 941 bp PCR product characteristic of Δ*slr0236*:Km^*r*^ chromosomes, and the absence of a 443 bp DNA band typical of WT (*slr0236*^+^) chromosomes]. The absence of WT (*slr0236*^+^) chromosome copies in Δ*slr0236*:Km^*r*^ mutants was confirmed by growing the cells for multiple generations in absence of Km to stop counter-selecting WT (*slr0236*^+^, i.e., Km^*S*^) chromosome copies. These Δ*slr0236*:Km^*r*^ cells grown in absence of Km harbored only Δ*slr0236*:Km^*r*^ mutant chromosomes, no WT (*slr0236*^+^, Km^*S*^) chromosomes (Figure 1A, see the 941 bp PCR product and the absence of a 443 bp PCR band). Together, these results show that *slr0236* is dispensable to cell viability.

In contrast, the attempted deletion of the *sll1545* gene invariably generated Km^*r*^ clones carrying two types of chromosomes, mutant (Δ*sll1545*:Km^*r*^) and WT (*sll1545*^+^) irrespectively of the duration of growth in the presence of Km used for selection (Figure 1B, see the 941 bp and 355 bp PCR products characteristic of, respectively, Δ*sll1545*:Km^*r*^ and *sll1545*^+^ chromosomes). Similarly, the attempted deletion of *sll1545* with the Δ*sll1545*:Sm^*r*^/Sp^*r*^ cassette generated clones harboring the two types of chromosomes, mutant Δ*sll1545*:Sm^*r*^/Sp^*r*^) and WT (*sll1545*^+^). Collectively, these results show that *sll1545* is essential to the photoautotrophic growth of *Synechocystis*.

In a parallel experiment, we also attempted to delete the *sll1545* gene in the Δ*slr0236*:Km^*r*^ mutant because we reasoned that the highly homologous Slr0236 and Sll1545 proteins could perform similar roles, which should be more affected, and thereby more identifiable, in the mutant combining the absence of Slr0236 and the depletion of Sll1545 than in the corresponding single mutants (lack of Slr0236 or depletion of Sll1545). Practically, we transformed the Δ*slr0236*:Km^*r*^ mutant with the Δ*sll1545*:Sm^*r*^/Sp^*r*^ cassette. As anticipated, the resulting Km^*r*^, Sm^*r*^/Sp^*r*^ mutant lacked the *slr0236*^+^ WT gene and retained the *sll1545*^+^ WT gene (Figure 1F, see the typical PCR DNA bands of Δ*slr0236*:Km^*r*^, Δ*sll1545*:Sm^*r*^/Sp^*r*^ and *sll1545*^+^ chromosomes, and the absence of the *slr0236*^+^ chromosome). These findings confirmed that *slr0236* and *sll1545* are, respectively, dispensable and crucial to cell growth in standard photoautotrophic conditions. The three mutants constructed in this study are designated as Δ*slr0236* (absence of *slr0236*), Δ*sll1545* (depletion of *sll1545*), and Δ*slr0236-sll1545* (combined absence of *slr0236* and depletion of *sll1545*).

### The Combination of the Deletion of *slr0236* and the Depletion of *sll1545* Confers a Light Sensitive Phenotype

**FIGURE 2:**
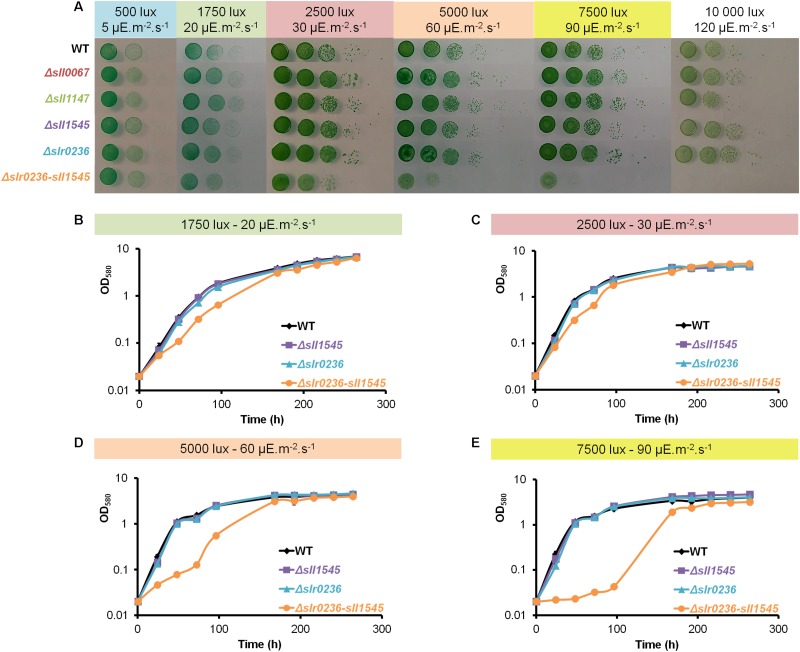
Influence of increasing light fluences on the growth of *Synechocystis* WT and mutants Δ*slr0236* (absence of *slr0236*), Δ*sll1545* (depletion of *sll1545*) and Δ*slr0236-sll1545* (combined absence of *slr0236* and depletion of *sll1545*). **(A)** Ten-fold serial dilutions of mid-log-phase cultures (initial OD_580_ = 0.1, i.e., 2.5 × 10^6^ cells mL^–1^) were spotted as 10 μL dots onto MM plates and incubated for 7 days at 30°C under the indicated light intensities prior to photography. **(B–E)** Typical growth curves (initial OD_580 *nm*_ = 0.02) of WT and mutant strains incubated in liquid MM under the indicated lights. All experiments were performed at least three times (error bars: standard deviation).

Cyanobacteria are often challenged by toxic ROS that are produced when photosynthesis generates more electrons than what is needed for CO_2_ assimilation (Naraimsamy et al., 2013). Consequently, to investigate the role of the Sll1545 and Slr0236 GSTs in the tolerance to light stress, we have tested the influence of various light intensities (1750, 2500, 7500, and 10000 lux) on the growth of our mutants in solid (Figure 2A) and liquid (Figures 2B–E) culture media. The two single mutants Δ*slr0236* (absence of *slr0236*) and Δ*sll1545* (depletion of *sll1545*) grew as fit as the WT strain and our other GST mutants (their construction and analysis will be published elsewhere) under all light fluences. These other mutants were showed here merely to avoid manipulating the image for not showing them. In contrast, the growth of the Δ*slr0236-sll1545* mutant was increasingly affected under increasing illumination, indicating that the combined absence of *slr0236* and depletion of *sll1545* decrease the tolerance to light.

### The Light-Sensitive Mutant Combining the Deletion of *slr0236* and the Depletion of *sll1545* Exposed to High-Light Undergoes a Transient Sequential Accumulation of ROS and Then of Reduced (GSH) and Oxidized (GSSG) Glutathione

**FIGURE 3:**
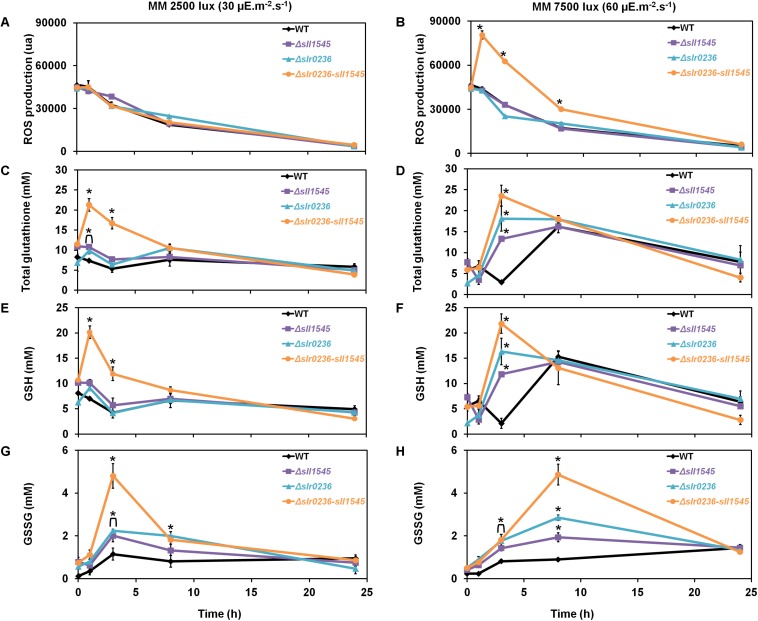
Kinetic analysis of the influence of light intensity on the abundance of reactive oxygen species (ROS) **(A,B)**, total glutathione **(C,D)**, GSH **(E,F)**, and GSSG **(G,H)** in *Synechocystis* WT and mutants Δ*slr0236*, Δ*sll1545*, and Δ*slr0236*-sll1545. Data are expressed as the mean ± SD (*n* = 3) and ^*^ indicates significant difference between mutant and WT (*t*-test, *P* < 0.05). Data are expressed as the mean ± SD (*n* = 3); ^*^ indicates significant difference between mutant and WT (*t*-test, *P* < 0.05) and for the sake of clarity, ⊏ indicates that the significant difference between mutant and WT (*t*-test, *P* < 0.05; symbolized by ^*^) applies to the grouped points.

To analyze the influence of high-light on the mutants Δ*slr0236* (absence of *slr0236*), Δ*sll1545* (depletion of *sll1545*), Δ*slr0236-sll1545* (combined absence of *slr0236* and depletion of *sll1545*) and the WT strain we measured the ROS content (Figures 3A,B), using the standard fluorescent probe DCHF-DA (Gomes et al., 2005). As GSTs are generally involved in glutathione-dependent reactions, we also measured the levels of the reduced (GSH), oxidized (GSSG) and total glutathione (GS_*total*_ = GSH + GSSG) (Figures 3G,H) using the relevant standard assay (Akerboom and Sies, 1981). In accordance with its light-sensitivity the Δ*slr0236-sll1545* mutant facing high light (7500 lux) rapidly (in 1 h) accumulated ROS (Figure 3B). Subsequently (at 3 h), the content of ROS decreased while the level of both GSH (the major form of glutathione) and GS_*total*_ increased (Figures 3B,D,F). Later (at 8 h) the decline of ROS has progressed and the decline of GSH and GS_*total*_ has begun, whereas the content of GSSG has increased (Figures 3B,D,F,H). In contrast, the two single mutants Δ*slr0236* and Δ*sll1545* exposed to high light showed (i) no accumulation of ROS and (ii) weaker glutathione responses, in agreement with their low susceptibility to high-light (Figures 3B,D,F,H). In a very different way, the WT strain exposed to high light exhibited a slight decrease (not increase) in GSH and GS_*total*_ (at 3 h), while the GSSG content was little affected, in agreement with the strong light tolerance of WT cells.

Collectively, these findings indicate that Slr0236 and Sll1545 normally operate in the protection against the ROS elicited by high-light, using a redox process that transforms reduced glutathione (GSH) in oxidized glutathione (GSSG). This process takes about 24 h. The transient changes in the levels of free GSH observed at 3 h: a decrease in the WT strain and an increase in mutants (higher in the Δ*slr0236-sll1545* mutant) are consistent with GST mediating the conjugation of GSH onto various types of ROS to detoxify them. The present data indicate that in *Synechocystis* the protection against ROS via their conjugation with GSH involves, at least in part, the Slr0236 and Sll1545 GST.

### The Deletion of *slr0236* and the Depletion of *sll1545* Decrease the Tolerance to Methylene Blue in a Cumulative Way

**FIGURE 4:**
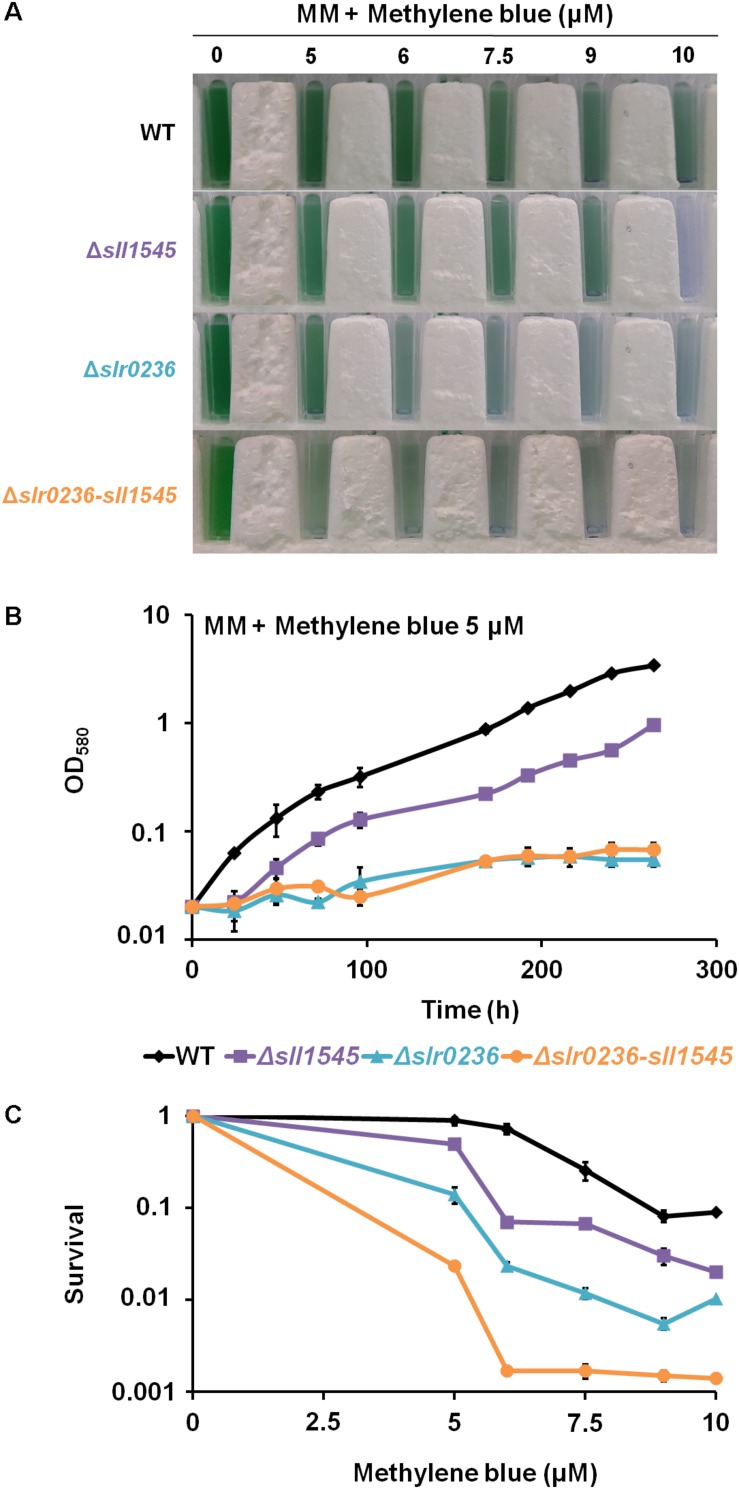
Influence of methylene blue on the growth and survival of *Synechocystis* WT and mutants Δ*slr0236*, Δ*sll1545*, and Δ*slr0236*-sll1545. **(A)** Ten milliliters of mid-log-phase cultures (OD_580_ = 0.1) were incubated in liquid MM with or without various concentrations of methylene blue for 72 h at 30°C under 2500 lux prior to transfer in spectrophotometric cuvettes and photography. **(B)** Typical growth curve of WT and mutants incubated in liquid MM with or without 5 μM methylene blue. **(C)** Typical survival of the WT and mutants exposed for 72 h to various doses of methylene blue. Data shown in **(B)** and **(C)** are expressed as the mean ± SD (*n* = 3).

As an excess of light can produce various type of ROS: singlet oxygen (^1^O_2_), superoxide anion (O_2_^∙–^), hydrogen peroxide (H_2_O_2_) and hydroxyl radical (OH^∙^), we have tested the influence of methylene blue (MB), which produces singlet oxygen ^1^O_2_ (Shao et al., 2013), on the growth and survival of the WT strain and the mutants Δ*slr0236* (absence of *slr0236*), Δ*sll1545* (depletion of *sll1545*) and Δ*slr0236-sll1545* (combined absence of *slr0236* and depletion of *sll1545*). The growth and survival of the mutants were more affected than those of the WT strains (Figure 4), showing that both Slr0236 and Sll1545 operate in the protection against MB. The higher tolerance of the Δ*sll1545* mutant as compared to Δ*slr0236* can be due to their difference in chromosome segregation. Δ*sll1545* cells in retaining wild-type allele of *sll1545* still possess Sll1545 proteins that can operate in resistance to MB. In contrast, Δ*slr0236* cells in harboring no WT allele of *slr023*6 have no Slr0236 protein that cannot participates to MB resistance. The test of the effects of various doses of MB on cell growth and survival showed that the Δ*slr0236-sll1545* strain was more stress-sensitive than the two corresponding single mutants Δ*slr0236* and Δ*sll1545* as shown above in the case of the high light stress. These data show that the combined depletion of *sll1545* and deletion of Δ*slr0236* elicit a cumulative decrease in the *Synechocystis* tolerance to high light or MB.

### The Δ*slr0236*, Δ*sll1545*, and Δ*slr0236-sll1545* Mutants Exposed to Methylene Blue Undergo a Transient Sequential Accumulation of ROS, and Then of Reduced and Oxidized Glutathione

**FIGURE 5:**
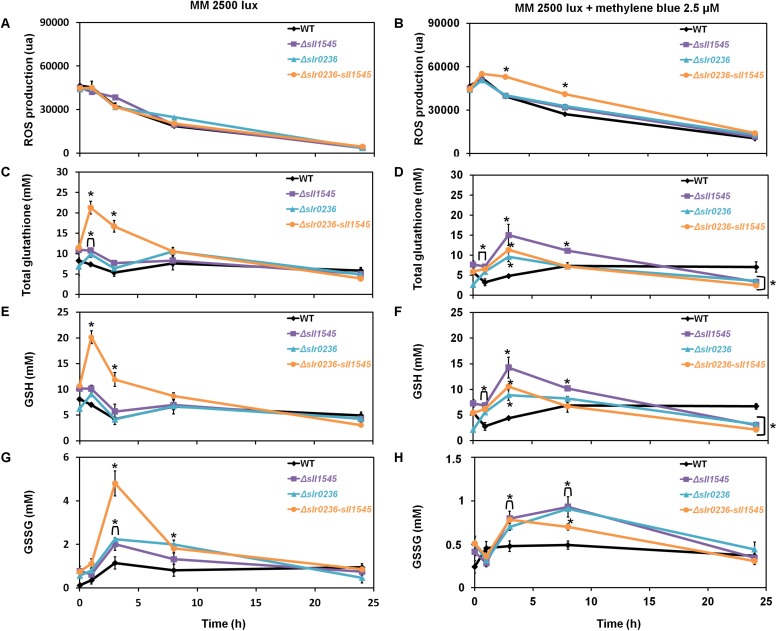
Kinetic analysis of the influence of methylene blue on the abundance of ROS **(A,B)**, total glutathione **(C,D)**, GSH **(E,F)** and GSSG **(G,H)** in WT, Δ*slr0236*, Δ*sll1545*, and Δ*slr0236*-sll1545 strains. Data are expressed as the mean ± SD (*n* = 3); ^*^ indicates significant difference between mutant and WT (*t*-test, *P* < 0.05) and for the sake of clarity, ⊏ indicates that the significant difference between mutant and WT (*t*-test, *P* < 0.05; symbolized by ^*^) applies to the grouped points.

The effects of methylene blue on the content of ROS and glutathione were similar but lower in amplitude than those triggered by the high-light stress (Figure 5), in agreement with the fact that singlet oxygens generated by MB (Shao et al., 2013) are not detected *per se* by the ROS-detecting DCHF-DA probe (Gomes et al., 2005) that we used. The similar difference in the glutathione responses triggered by high light (strong effect) and methylene blue (lower impact) stresses could indirectly result from this explanation. The mutants displayed a rapid (in 1 h) and low increase in ROS (Figure 5B). Then (at 3 h), the ROS started their continuous decline while both GSH and GS_*total*_ increased (Figures 5B,D,F). Later (at 8 h) the levels of ROS, GSH, and GS_*total*_ have declined, whereas the GSSG has increased before returning to a lower (unstressed) level (around 24 h) (Figures 5B,D,F,H). In contrast, WT cells exposed to methylene blue exhibited a slight decrease (at 1 h) in GSH and GS_*total*_ levels, and a slight increase in GSSG, before returning to unstressed levels. Collectively, these finding indicate that Slr0236 and Sll1545 normally operate in the GSH-dependent protection against the ROS elicited by methylene blue.

### The Combined Deletion of *slr0236* and Depletion of *sll1545* Increase the Tolerance to Menadione Accompanied by a Transient Accumulation of GSH and GSSG

**FIGURE 6:**
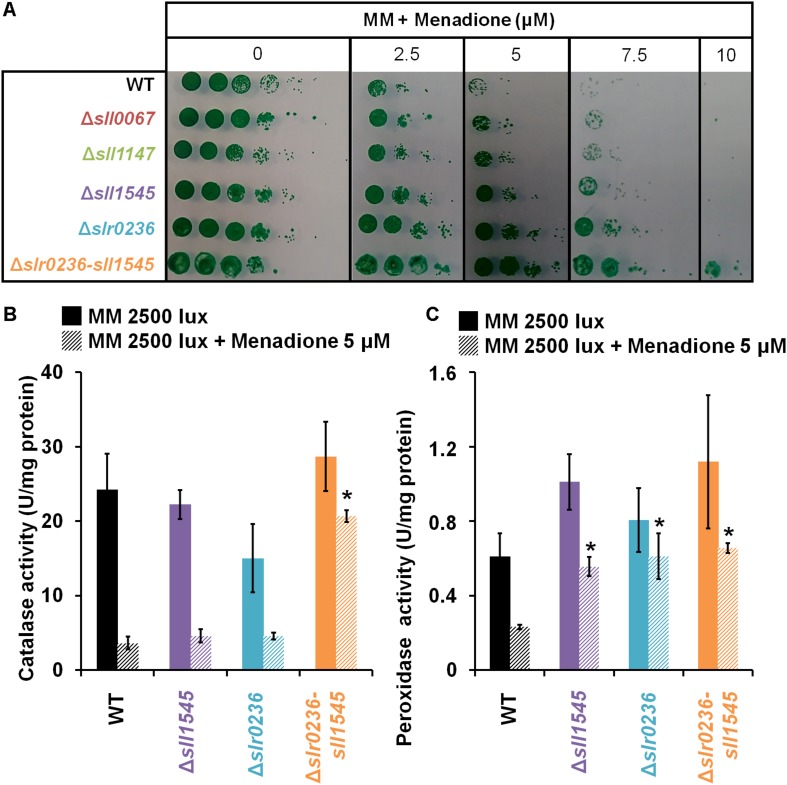
Influence of menadione treatment on the growth, and catalase and peroxidase activities, of *Synechocystis* WT and mutants Δ*sll1545*, Δ*slr0236* and Δ*slr0236-sll1545*. **(A)** Ten-fold serial dilutions of mid-log-phase cultures (initial OD_580 *nm*_ = 0.1) were spotted as 10 μL dots onto MM plates with or without menadione and incubated for 7 days at 30°C under 2500 lux, prior to photography. Histogram plot representation of **(B)** catalase and **(C)** peroxidase activities of cells before or after a 1 h menadione treatment. Data are expressed as the mean ± SD (*n* = 3) and ^*^ indicates significant difference between mutant and WT (*t*-test, *P* < 0.05).

**FIGURE 7:**
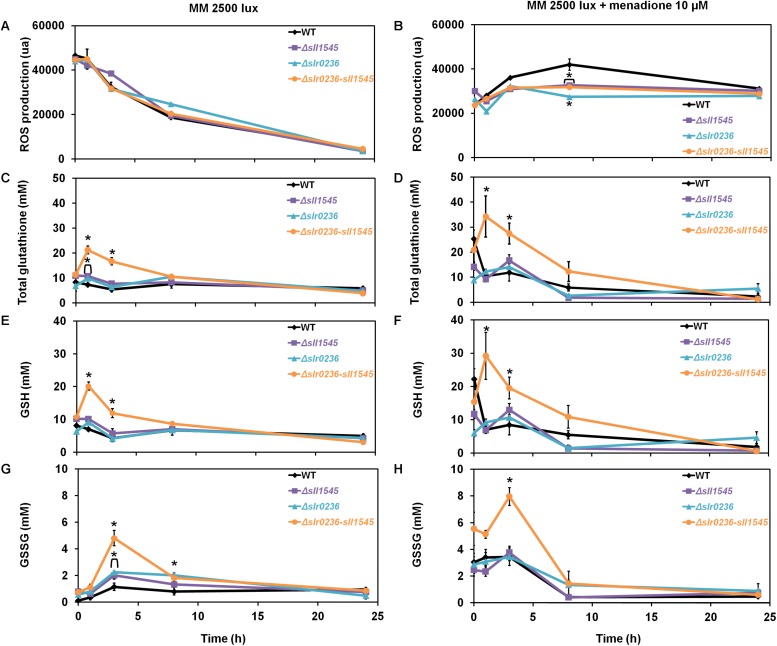
Kinetic analysis of the influence of menadione on the abundance of reactive oxygen species (ROS) **(A,B)**, total glutathione **(C,D)**, GSH **(E,F)**, and GSSG **(G,H)** in WT, Δ*slr0236*, Δ*sll1545*, and Δ*slr0236*-sll1545 strains. Data are expressed as the mean ± SD (*n* = 3); ^*^ indicates significant difference between mutant and WT (*t*-test, *P* < 0.05) and for the sake of clarity, ⊏ indicates that the significant difference between mutant and WT (*t*-test, *P* < 0.05; symbolized by ^*^) applies to the grouped points.

As part of the *in vivo* analysis of the physiological role of the Slr0236 and Sll1545 GST, we have also tested the influence of menadione, which generates superoxide anions (O_2_^∙–^) (Moirangthem et al., 2014), on the growth (on solid medium) of the WT strain and the mutants Δ*slr0236*, Δ*sll1545* and Δ*slr0236-sll1545* (Figure 6A). The mutants Δ*sll1545*, Δ*slr0236*, and Δ*slr0236-sll1545*, in that order, appeared to be more resistant than the WT strain. This finding indicates that the protein Sll1545 and Slr0236 have a negative influence on the protection against menadione. The different level of menadione resistance exhibited by the mutants could be explained by the above-mentioned absence of *slr0236* in Δ*slr0236* cells, the depletion of *sll1545* in Δ*sll1545* cells, and their cumulative influence on MB resistance. As controls, we have tested other GST deleted mutants (their construction and analysis will be published elsewhere). That they behaved as the WT strain indicate that not all *Synechocystis* GST operate (directly or indirectly) in the protection against menadione, thereby emphasizing GST selectivity. In the mutants Δ*sll1545*, Δ*slr0236*, and Δ*slr0236-sll1545*, the presence of menadione prevented the steadily decline of ROS triggered by light (Figures 7A,B). In the (sensitive) WT strain, menadione triggered a rapid (at 1 h) and temporary decline of GSH, indicative of the GSH-mediated ROS detoxification. In contrast, in the Δ*slr0236-sll1545* mutant, the best menadione resistant strain, menadione slightly amplified the light-induced transient accumulation of GSH (at 1 h) and then of GSSG (at 3 h) (Figures 7D,F,H). This glutathione response was not really observed in the single mutants Δ*sll1545* and Δ*slr0236*, in spite of their (lower) menadione resistance. Collectively these data indicate that in addition to GSH other players contribute to the menadione resistance of the mutants Δ*sll1545*, Δ*slr0236*, and Δ*slr0236-sll1545*.

### The Increased Menadione Resistance of the Mutants Δ*slr0236*, Δ*sll1545*, and Δ*slr0236-sll1545* Is Due, at Least in Part, to Their Higher Level of Catalase and/or Peroxidase Activity

To search for players involved in the high menadione resistance of the mutants, we used standard protocols to measure the catalase and peroxidase activities of the *Synechocystis* bifunctional catalase/peroxidase enzyme (Tichy and Vermaas, 1999) because menadione generated superoxide anions (O_2_^∙–^) are detoxified by superoxide dismutase, which produces dioxygen (O_2_) and hydrogen peroxide (H_2_O_2_), and catalase, which transforms H_2_O_2_ to water and dioxygen. The strains were incubated before or after a 1 h exposure to 5 μM menadione (a concentration well discriminating the level of menadione tolerance). In all strains both catalase and peroxidase activities appeared to be decreased by menadione (Figures 6B,C). The decline was highest in the menadione sensitive WT strain, whereas it was lowest in the highest menadione-resistant Δ*slr0236-*sll1545 mutant. In single mutants Δ*sll1545* and Δ*slr0236* with an intermediate level of menadione tolerance, the peroxidase activity was less decreased by menadione than in the menadione-sensitive WT strain. Collectively, these data indicate that the higher level of catalase and/or peroxidase activities of the mutants Δ*slr0236*, Δ*sll1545* and, particularly, Δ*slr0236-sll1545* contribute to their increased menadione resistance. These findings also indicate that Slr0236 and Sll1545 play a negative (direct or indirect) role on the catalase and/or peroxidase activities of *Synechocystis*.

### The Δ*sll1545* Mutant Is Sensitive to H_2_O_2_, Unlike the Mutants Δ*slr0236* (Insensitive) and Δ*slr0236-sll1545* (Resistant)

**FIGURE 8:**
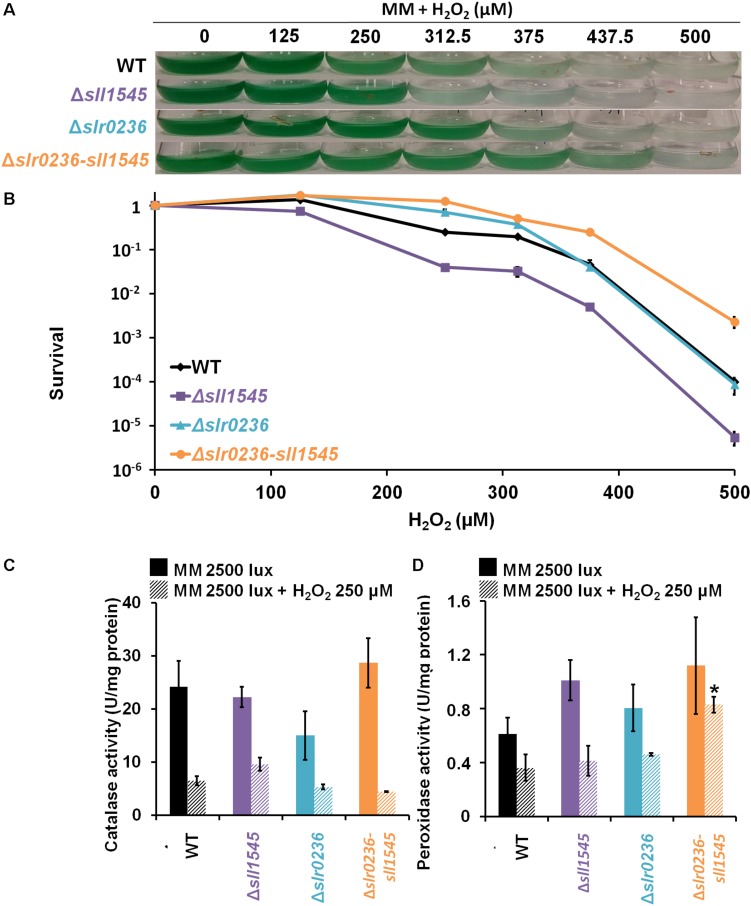
Influence of hydrogen peroxide on the growth, survival and catalase-peroxidase activities of *Synechocystis* WT and mutant strains Δ*sll1545*, Δ*slr0236*, and Δ*slr0236-sll1545*. **(A)** Ten milliliters of mid-log-phase (initial OD_580 *nm*_ = 0.1) were incubated in liquid MM at 30°C under 2500 lux with or without hydrogen peroxide for 72 h prior to photography of the Erlenmeyer flasks. **(B)** Typical survival of the WT and mutants after 72 h-exposure to hydrogen peroxide. Histogram plot representation of catalase **(C)** and peroxidase **(D)** activities of cells before and after a 1 h H_2_O_2_ treatment. Data are expressed as the mean ± SD (*n* = 3) and ^*^ indicates significant difference between mutant and WT (*t*-test, *P* < 0.05).

We have also tested the influence of H_2_O_2_ on the growth and survival of the WT, Δ*slr0236*, Δ*sll1545*, and Δ*slr0236-sll1545* strains (Figures 8A,B). In comparison to the WT strain, the mutant Δ*sll1545* appeared to be sensitive, in agreement with the GSH-dependent peroxidase activity of Sll1545 protein described *in vitro* (Pandey et al., 2017). In contrast, the mutant Δ*slr0236* behaved as the WT strain, suggesting that Slr0236 is not involved in the protection against H_2_O_2_. The H_2_O_2_ resistance of the Δ*slr0236-sll1545* mutant is not easy to explain as it combines the depletion of *sll1545*, a positive player in H_2_O_2_ detoxication, with the absence of *slr0236*, a neutral player.

### Influence of H_2_O_2_ on the Levels of ROS and Glutathione in the Mutants Δ*slr0236*, Δ*sll1545*, and Δ*slr0236-sll1545*

**FIGURE 9:**
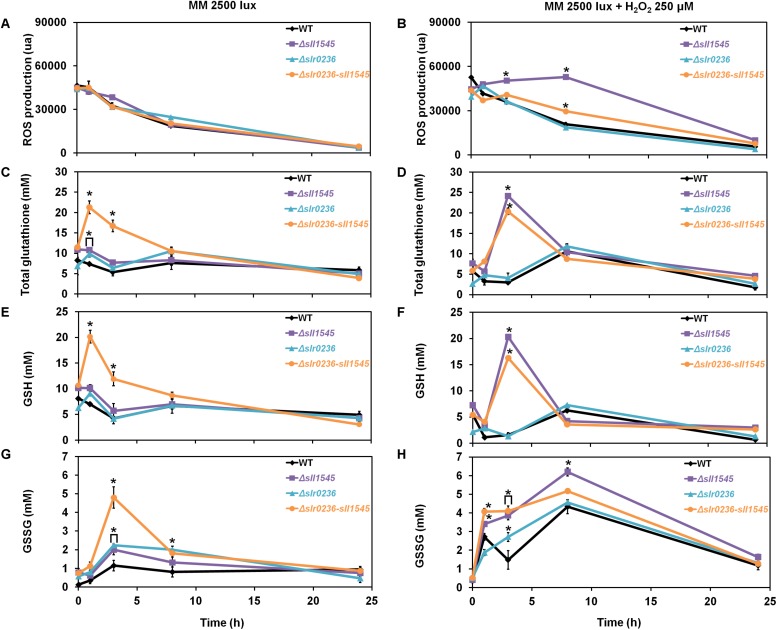
Kinetic analysis of the influence of hydrogen peroxide on the abundance of reactive oxygen species (ROS) **(A,B)**, total glutathione **(C,D)**, GSH **(E,F)**, and GSSG **(G,H)** in WT, Δ*slr0236*, Δ*sll1545*, and Δ*slr0236*-sll1545 strains. Data are expressed as the mean ± SD (*n* = 3); ^*^ indicates significant difference between mutant and WT (*t*-test, *P* < 0.05) and for the sake of clarity, ⊏ indicates that the significant difference between mutant and WT (*t*-test, *P* < 0.05; symbolized by ^*^) applies to the grouped points.

In response to 250 μM H_2_O_2_, the mutant Δ*sll1545* exhibited a high level of ROS that lasted during 8 h, a time during which we noticed a low and transient decrease of GSH (at 1 h) followed by a strong and temporary increase (at 3 h) of GSSG, followed by a strong and transient increase of GSSG (peak at 8 h). These findings (Figure 9) are consistent with the H_2_O_2_ sensitivity of Δ*sll1545* cells (Figure 8).

In contrast, the WT and Δ*slr0236* strains exhibited no ROS accumulation (Figures 9B,D,F,H), in agreement with their low sensitivity to this dose (250 μM) of H_2_O_2_ (Figure 8). These WT and Δ*slr0236* cells showed only minor changes of their level of GSH (low and transient decrease at 1 h followed by a small and temporary increase at 8 h) and GSSG (two low and transitory increases at 1 and 8 h, intersected by a low and temporary decrease at 3 h).

In the H_2_O_2_-treated Δ*slr0236-sll1545* cells, the levels of ROS and glutathione were not increased above the level driven by light alone (Figures 9A,B). The responses of GSH and GSSG were slightly modified only in term of kinetic, possibly contributing to the H_2_O_2_ resistance of these cells. At 1 h we noticed a H_2_O_2_-mediated concomitant decrease of GSH and increase of GSSG indicative of ROS detoxification. At 3 h, instead of the GSSG accumulation peak triggered by light alone, we observed a H_2_O_2_-mediated peak of GSH accumulation and a little decrease in GSSG. Then, at 8 h, we observed a normal level of only GSH, not GSSG which returned to normal level only after 20 h. These findings are not sufficient to explain the difference in the H_2_O_2_ sensitivity/tolerance of these strains; other players need to be searched for.

### The Increased H_2_O_2_ Resistance of the Δ*slr0236-sll1545* Mutant Is Due, at Least in Part, to Its Higher Level of Peroxidase Activity

To test the influence of the catalase and peroxidase in the H_2_O_2_ tolerance, we measured these enzyme activities in the Δ*sll1545*, Δ*slr0236*, and Δ*slr0236-sll1545* mutants and the WT strain incubated before or after a 1 h exposure to 250 μM H_2_O_2_ (an effective but none-lethal concentration). Both catalase and peroxidase activities of the WT strain and the Δ*sll1545* and Δ*slr0236* mutants were strongly decreased by H_2_O_2_ (Figures 8C,D). In contrast, the catalase activity of the Δ*slr0236-sll1545* mutant was declined by H_2_O_2_, whereas its peroxidase activity was stable. This finding suggests that the H_2_O_2_-insensitive efficient peroxidase activity of the mutant Δ*slr0236-sll1545* contribute to its high resistance to H_2_O_2_.

## Discussion

It is important to analyze the glutathione (GSH) and the GSTs in cyanobacteria, the environmentally crucial photosynthetic prokaryotes (Cassier-Chauvat and Chauvat, 2018), because these microorganisms are regarded as having developed GSH and GSTs to cope with the ROS often produced by their active photosynthesis (William Schopf, 2011). While low changes of ROS levels can function in signaling (Schieber and Chandel, 2014) large changes are merely toxic (Yadav, 2010; Noctor et al., 2012). Cyanobacteria also have valuable biotechnological potentials (ecological production of chemicals), which are often hampered by a lack of knowledge of their responses to stresses that limit the engineering of powerful cell factories (for a discussion, of this fact see Jones, 2014; Cassier-Chauvat et al., 2016). Furthermore, many of the effective anti-oxidant processes that likely emerged in cyanobacteria have been conserved and complexified in higher plants and mammals, which are more complex to study than cyanobacteria, in possessing various types of tissue. Moreover, plants and mammals often possess large families of enzymes that renders difficult the analysis of the selectivity/redundancy of these enzymes. For example, the model plant *Arabidopsis thaliana* has about 55 GSTs (Gallé et al., 2018) whereas the best-studied cyanobacterium *Synechocystis* PCC 6803 (*Synechocystis*), possesses only six GSTs.

In this study, we have analyzed *in vivo* the role of the GST Sll1545 to extend previous *in vitro* studies (Pandey et al., 2017), and of Slr0236 because we found that it shares the greatest sequence identity (about 32%) with Sll1545.

We demonstrate that the *sll1545* gene is essential for the photoautotrophic growth of *Synechocystis* whereas *slr0236* is dispensable, and that the combination of the deletion of *slr0236* and the depletion of *sll1545* decreases the photo-tolerance of *Synechocystis*. The finding that the phenotype of the Δ*slr0236-sll1545* mutant is more severe that the phenotypes of the two single mutants Δ*slr0236* and Δ*sll1545* taken individually, suggest that Sll1545 and Slr0236 operates in similar but not completely overlapping light resistance processes. Consistently with its light-sensitive phenotype, only the Δ*slr0236-sll1545* mutant exposed to high light (Figure 2), transiently accumulates ROS (at 1 h) and then reduced and oxidized forms of glutathione in that order (at 3 h and 8 h, respectively, Figure 3F). These data indicate that to detoxify high-light-triggered ROS, cells accelerate the synthesis of reduced GSH, which is oxidized in glutathione disulfide (GSSG) upon detoxification. Our findings are consistent with other studies in *A. thaliana* (Queval et al., 2009). An increase in GSH synthesis has been also observed in *Chlamydomonas reinhardtii* challenged by high-light (Lin et al., 2018).

To get a comprehensive view of the role of Sll1545 and Slr0236, the mutants Δ*slr0236*, Δ*sll1545*, and Δ*slr0236-sll1545* were also challenged with methylene blue, menadione and H_2_O_2_. We found that the deletion of *slr0236* and the depletion of *sll1545* decrease the tolerance to methylene blue in a cumulative way. As light-stressed cells, methylene-blue challenged cells temporarily accumulated ROS, and then GSH and GSSG in that order (Figure 5) indicating that Slr0236 and Sll1545 operate in the GSH-dependent detoxication of the ROS elicited by light or methylene blue.

Unlike what was observed with high-light and methylene blue, the mutants Δ*slr0236*, Δ*sll1545*, and Δ*slr0236-sll1545* mutants appeared to be more resistant to menadione that the WT strain (Figure 7). The single mutants Δ*sll1545* and Δ*slr0236* exhibited no menadione-induced change in glutathione level, whereas the Δ*slr0236-sll1545* mutant, the most menadione resistant strain, transiently accumulated GSH and then GSSG (Figure 7). These data indicate that besides GSH other players contribute to the menadione resistance of the mutants Δ*sll1545*, Δ*slr0236*, and Δ*slr0236-sll1545*. Indeed, we found that the increased menadione resistance of the mutants Δ*slr0236*, Δ*sll1545*, and Δ*slr0236-sll1545* is due, at least in part, to their higher level of catalase and/or peroxidase activity (Figure 6). In turn, these findings suggest that in WT cells the Sll1545 and Slr0236 proteins negatively influence (directly or indirectly) the catalase and/or peroxidase activity. Finally, we also challenged the mutants Δ*slr0236*, Δ*sll1545*, and Δ*slr0236-sll1545* with hydrogen peroxide. In response to 250 μM H_2_O_2_, the WT and Δ*slr0236* strains showed no ROS accumulation (Figure 9) and only minor changes of their levels of GSH, in agreement with these cells being weakly affected by this H_2_O_2_ dose (Figure 8). In contrast, the Δ*sll1545* mutant exhibited a high level of ROS that lasted 8 h, a time during which we noticed mainly a strong and transient increase of GSH at 3 h, followed by a strong and temporary increase of GSSG at 8 h. These findings (Figure 9) are consistent with the H_2_O_2_ sensitivity of Δ*sll1545* cells (Figure 8). In the H_2_O_2_-treated Δ*slr0236-sll1545* cells, the levels of ROS and glutathione were not increased above the level driven by light alone (Figure 9). The responses of GSH and GSSG were slightly modified only in term of kinetic, possibly contributing to the H_2_O_2_ resistance phenotype of these cells. Finally, we found that the active H_2_O_2_-insensitive peroxidase activity of the mutant Δ*slr0236-sll1545* contribute to its high resistance to H_2_O_2_. This may contribute to the difference in H_2_O_2_-tolerance of the single mutant Δ*sll1545* (sensitive) and the Δ*slr0236-sll1545* mutant (resistant).

In conclusion, we report the first *in vivo* analysis of cyanobacterial GSTs, namely Sll1545 and Slr0236, two of the six GSTs of the widely studied model *Synechocystis* PCC 6803. Our results indicate that these two homologous GST operate with glutathione and/or the catalase/peroxidase activities (directly or indirectly) to protect cells against photo-oxidative stresses triggered by high light, H_2_O_2_, methylene blue and menadione, using as yet uncharacterized molecular mechanisms. These findings are interesting for readers studying bacterial stress responses, including in the view of increasing the stress resistance of cyanobacteria that are engineered for the photoproduction of toxic chemicals. Furthermore, our data may facilitate the analysis of the multiple GSTs of complex organisms (plants, mammals) with multiple cell types and tissues.

## Materials and Methods

### Bacterial Strains, Growth, and Stress Assays

*Escherichia coli* TOP10 (Invitrogen) used for gene manipulation was grown on LB at 37°C. Antibiotic selections were performed with ampicillin (Amp) 100 μg mL^–1^, kanamycine (Km) 50 μg mL^–1^, or both streptomycin (Sm) 25 μg mL^–1^ and spectinomycin (Sp) 75 μg mL^–1^.

*Synechocystis* PCC 6803 (*Synechocystis*) was routinely grown at 30°C in liquid mineral medium (MM), i.e., BG11 medium (Rippka et al., 1979) enriched with 3.78 mM Na_2_CO_3_ (Jittawuttipoka et al., 2013), under continuous agitation (140 rpm) and white light (2500 lux; 30 μE m^–2^ s^–1^) at 30°C unless stated otherwise. Antibiotic selections were performed with Km 50 μg mL^–1^ or both Sm 5 μg mL^–1^ and Sp 5 μg mL^–1^. For stress analyses in *Synechocystis*, mid-exponential phase cultures (OD_580 *nm*_ = 0.3 to 0.8) were adjusted to OD_580_ = 0.02 (5.10^5^ cells mL^–1^) for growth analysis or OD_580_ = 0.1 (2.5 × 10^6^ cells mL^–1^) for survival analysis and subsequently incubated for various durations in liquid or on solid media containing the indicated noxious agents prior to measuring OD_580_ or photographing the flasks culture or plates. For survival analysis cells were serially diluted in MM, spread on MM solidified with 1% agar (Difco) and incubated during 5–7 days under standard conditions (2500 lux; 30°C) before counting.

### Construction of the DNA Cassette for Targeted Deletion of the *sll1545* and *slr0236* Genes

*Synechocystis* DNA regions (about 300 bp in length) flanking the studied protein coding sequence (CS) were independently amplified by PCR, using specific oligonucleotides primers (Supplementary Table S2). These two DNA regions were joined by standard PCR-driven overlap extension (Heckman and Pease, 2007) in a single DNA segment harboring a *Sma*I restriction site in place of the studied CS. After cloning in pGEMT (Promega) the resulting plasmids (Supplementary Table S1) were opened at the unique *Sma*I site where we cloned the Km^*r*^ cassette (a *Hin*cII fragment of the commercial pUC4K plasmid) in the same orientation as the coding sequence it replaced. The Δ*sll1545*:Sm^*r*^/Sp^*r*^ cassette was generated by replacing the Km^*r*^ DNA cassette by the Sm^*r*^/Sp^*r*^ DNA cassette from pFC1 plasmid (Mermet-Bouvier and Chauvat, 1994) using a DNA assembly strategy (NEBuilder HiFi DNA Assembly Master Mix, New England BioLabs).

The resulting deletion cassettes Δ*sll1545*:Km^*r*^, Δ*slr0236*:Km^*r*^ and Δ*sll1545*:Sm^*r*^/Sp^*r*^ were verified by PCR and nucleotide sequencing (Mix2Seq Kit, Eurofins Genomics) before and after transformation (Labarre et al., 1989) to *Synechocystis* WT cells (to generate the Δ*sll1545*:Km^*r*^ and Δ*slr0236*:Km^*r*^ mutants) or to the Δ*slr0236*:Km^*r*^ mutant (to generate the Δ*sll1545-slr0236* mutant).

### Cell Culture and Assay of the Reduced (GSH) and Oxidized (GSSG) Forms of Glutathione

All reagents were purchased from Sigma-Aldrich. Fifty milliliters of exponentially growing cultures were diluted twofold down to OD_580_ = 0.4, and incubated for various durations under white light (2500, 5000, or 7500 lux) or at 2500 lux in the presence of various agents as indicated. Cells were rapidly collected by filtration on a 0.45 μm cellulose membrane (Millipore) under light; re-suspended in 1 mL of acidic extraction phosphate buffer [100 mM KH_2_PO_4_/K_2_HPO_4_; 1 mM EDTA; 5% (w/v) 5-sulfosalicylic acid (SSA)]; disrupted by a three freezing-thawing cycles in liquid nitrogen and hot water bath and strong mixing (Vibrax VXR, Ika) for 10 min at 4°C; prior to centrifugation (14,000 rpm, 4°C, 5 min) to eliminate unbroken cells and membranes. Cell extracts containing glutathione (GSH + GSSG) were purified by a 20 min centrifugation at 14,000 rpm at 4°C through a filter (Amicon Ultra – 0.5 mL 30K; Millipore) to eliminate proteins larger than 30 kDa. Filtrates were stored at −80°C until use. Before GSSG assay, 100 μL of filtrate were treated with 2 μL of neat 2-vinylpyridine for 1 h on ice to block reduced GSH and then with 2 μL of fourfold diluted triethanolamine solution. For assays, 10 μL untreated filtrate samples (total glutathione assay) and 20 μL treated samples (oxidized GSSG assay) were loaded on a UV-compatible 96-well plate (Greiner bio-one). Then, a first reaction mixture containing yeast GR at final concentration 1.25 U/mL in phosphate buffer (100 mMKH_2_PO_4_/K_2_HPO_4_ buffer and 1 mM EDTA, pH 7.5) was prepared and then distributed to each well. A second reaction mixture containing 0.2 mM DTNB [5,5′-dithiobis-(2-nitrobenzoic acid)] and 0.3 mM NADPH in phosphate buffer was automatically added in each well by a microplate reader (ClarioStar; BMG Labtech). The reaction was immediately followed by measuring for 1 min at 30°C the absorption at 412 nm of the yellow TNB (5′-thio-2-nitrobenzoic acid) product (Akerboom and Sies, 1981). In parallel, standard curves prepared with various concentrations of GSH (50 to 200 μM) or GSSG (10 to 250 μM) were used to calculate the GSSG (oxidized) and total glutathione (GSSG + reduced GSH) using the *Synechocystis* cell volume value of 1.2.10^–11^ mL (Mazouni et al., 2004). The GSH content was calculated by subtracting the GSSG content from the total glutathione content.

### Assay of the Cellular Reactive Oxygen Species (ROS) Content

The level of ROS was assayed with the fluorescent reactant 2′,7′-dichlorodihydrofluorescein diacetate (DCHF-DA; Sigma-Aldrich) that is converted into the non-fluorescent derivative DCHF by cellular esterases (Gomes et al., 2005). Subsequently, DCHF can be oxidized to the highly fluorescent DCF probe by intracellular ROS (hydrogen peroxide H_2_O_2_, hydroxyradical OH^∙^ and peroxyradical ROO^∙^). ROS triggered by the indicated treatments was evaluated on exponentially growing cultures calibrated to OD_580_ = 0.3. Then, DCHF-DA solubilized in ethanol was added (5 μM final concentration) and the cell suspensions were incubated in the dark to prevent auto-oxidation of the probe. The signal fluorescence (λ_*exc*_ = 485 nm and λ_*em*_ = 520 nm) was measured with a microplate spectrofluorimeter (CLARIOstar; BMG LABTECH), and normalized on the basis of the OD_580_.

### Catalase-Peroxidase Activity Assay

Cells treated for 1 h under the indicated conditions were broken with an Eaton press and cell free protein protein extracts were prepared as previously described (Mermet-Bouvier and Chauvat, 1994). Then, catalase activity was determined spectrophotometrically by monitoring the rate of H_2_O_2_ decomposition at 240 nm (ε_240_ = 43.6 M^–1^ cm^–1^) (Moirangthem et al., 2014) in reaction mixtures containing 10 mM Tris–HCl pH 7.5 buffer and 10 mM H_2_O_2_. Peroxidase activity was determined spectrophotometrically by following the rate of pyrogallol oxidation at 430 nm (ε_430_ = 2,47 mM^–1^ cm^–1^) (Moirangthem et al., 2014) in reaction mixtures containing 10 mM Tris–HCl pH 7.5 buffer, 20 mM pyrogallol and 0.1 mM H_2_O_2_. Catalase and peroxidase activities were expressed in units per mg of protein measured using Bradford assay (Biorad). One unit corresponds the decomposition of 1 μmol of H_2_O_2_ or the oxidation of 1 μmol of pyrogallol in 1 min, respectively.

## Data Availability

The raw data supporting the conclusions of this manuscript will be made available by the authors, without undue reservation, to any qualified researcher.

## Author Contributions

CC-C and FC conceived the project. XK, CC-C, and FC conceived the experiments and analyzed the data. XK performed the experiments. XK, CC-C, and FC wrote the manuscript. CC-C agreed to serve as the author responsible for contact and ensures communication.

## Conflict of Interest Statement

The authors declare that the research was conducted in the absence of any commercial or financial relationships that could be construed as a potential conflict of interest.

## Footnotes

1 http://genome.microbedb.jp/cyanobase/
