# Supplementary material for: First in vivo Evidence That Glutathione-S-Transferase Operates in Photo-Oxidative Stress in Cyanobacteria
Source: Front Microbiol. 2019 Aug 13;10:1899. doi: 10.3389/fmicb.2019.01899 (PMC6700277; doi:10.3389/fmicb.2019.01899)
Supplement: Supplementary file 1 [file Table_1.docx]

First *in vivo* evidence that glutathione-S-transferase operates in photo-oxidative stress in cyanobacteria

| **Supplementary Table S1 – List and characteristics of the plasmids used in this study** | | |
| --- | --- | --- |
| **Plasmid** | **Relevant features** | **Reference** |
| **pGEMT** | AT overhang cloning vector Amp^R^ | Promega |
| **pUC4K** | Source of the Km^R^ marker gene | Pharmacia |
| **pFC1** | Source of the Sm^R^/Sp^R^ marker gene | (Mermet-Bouvier and Chauvat, 1994) |
| **pGEMT-AB*sll1545*** | pGEMT with the *sll1545* gene and its flanking sequences, where most of the *sll1545* coding sequence (from the 5^th^ bp downstream of the ATG codon down to 5^th^ bp upstream to the TAG stop codon) was replaced by a *Sma*I restriction site | This study |
| **pGEMT-Δ*sll1545*::Km^R^** | pGEMT-AB*sll1545* with the Km^R^ marker inserted in the unique *Sma*I site | This study |
| **pGEMT-Δ*sll1545*::Sm^R^/Sp^R^** | pGEMT-AB*sll1545* with the Sm^R^/Sp^R^ marker inserted in the unique *Sma*I site | This study |
| **pGEMT-AB*slr0236*** | pGEMT with the *slr0236* gene and its flanking sequences, where most of the *slr0236* coding sequences (from the 26^th^bp downstream of the ATG codon down to the 22^th^bp upstream the TAG stop codon up) was replaced by a *Sma*I restriction site | This study |
| **pGEMT-Δ*slr0236*::Km^R^** | pGEMT-AB*slr0236* with the Km^R^ marker inserted in the unique *Sma*I site | This study |
| **pGEMT-Δ*slr0236*::Sm^R^/Sp^R^** | pGEMT-AB*slr0236* with the Sm^R^/Sp^R^ marker inserted in the unique *Sma*I site | This study |

**Supplementary Table S2 – List and characteristics of the PCR primers used in this study**

| **Name** | | **Sequence (5’→3’)** | **Purpose** |
| --- | --- | --- | --- |
|  | | | |
| **Amplification of the Km^r^ cassette** | | | |
| **KmHincII-FW** | GGCGCTGAGGTCGACCTCGTGAAGAAG | | Amplification of the Km^r^ cassette to be cloned as a *Hinc*II fragment |
| **KmHincII-RV** | ACCTGCAGGGGGTCGACGGAAAGCCAC | |  |
| **Amplification of a part of the Km^r^ cassette** | | | |
| **Km-FW (3)** | GGTGTTATGAGCCATATTCAACGGG | | Amplification of Km^r^ cassette to verify targeted gene replacement  and assay chromosome segregation |
| **Km-RV (4)** | GGGAAGATGCGTGATCTGATCCTTC | |  |
| **Amplification of a part of the Sm^r^/Sp^r^ cassette** | | | |
| **Sm/Sp-FW (5)** | ATCTCGAACCGACGTTGCTG | | Amplification of Sm^r^/Sp^r^ cassette to verify targeted gene replacement  and assay chromosome segregation |
| **Sm/Sp-RV (6)** | CCGACTACCTTGGTGATCTC | |  |
| **Deletion of *slr0236*** | | | |
| **R0236-FWa** | CTGGGAACAGCGGAAGTG | | Amplification of the *slr0236* downstream region and simultaneous introduction of a *Sma*I site |
| **R0236-RVa** | **AGACCCGGGGGCAAC**AGCAACAACATCCTA | |  |
| **R0236-FWb** | **GTTGCCCCCGGGTCT**AGGTTAGGGATCGTT | | Amplification of the *slr0236* upstream region and simultaneous introduction of a *Sma*I site |
| **R0236-RVb** | TTGGAAAACAAGCAGAAATTAAC | |  |
| **SLR0236-FW (1’)** | GTGTCCATGCCCTGCAACTTAAACC | | Amplification of a part of *slr0236* to verify chromosome segregation |
| **SLR0236-RV (2’)** | CGTTAGGTCTGCTATGGTCAACTGG | |  |
| **Deletion of *sll1545*** | | | |
| For simple mutant Km^r^ construction | | | |
| **L1545-FWa** | TGGTCTAAATTTTTAATTTCCCACT | | Amplification of the downstream region of the *sll1545* coding sequence and simultaneous introduction of a *Sma*I site |
| **L1545-RVa** | **TTGTTCCCGGGTACTA**AGCATGGTAGGAATTTTAG | |  |
| **L1545-FWb** | **TAGTACCCGGGAACAA**AGTAGGTGGTTTTCAACAA | | Amplification of the upstream region of the *sll1545* coding sequence and simultaneous introduction of a *Sma*I site |
| **L1545-RVb** | CCTGGATTGGGCCTTATTTG | |  |
| **SLL1545-FW (1)** | GGACATTGTCATTGCAGATTCGACG | | Amplification of a part of *sll1545* to verify chromosome segregation |
| **SLL1545-RV (2)** | CAGAGTTAAAGCCTCCAAGTCCTGA | |  |
| For double mutant Km^R^ and Sm^r^/Sp^r^ construction | | | |
| **Vect1545-FW** | GGGAACAAAGTAGGTGG | | Amplification of pGEMt-AB*sll1545* |
| **Vect1545-FV** | GGGTACTAAGCATGGTAGG | |  |
| **Sm/Sp-A1545-FW** | TCCTACCATGCTTAGTACCCGCGCTCACGCAACTGGTCCA | | Amplification of Sm^r^/Sp^r^ cassette from pFC1 plasmid |
| **Sm/Sp-B1545-RV** | **AAACCACCTACTTTGTTCCC**CTCCCAATTTGTGTAGGGCT | |  |
| FW: forward ; RV: reverse ; Numbers [(1); (2); (1’); (2’); (3); (4); (5) and (6)]: correspond to primers represented as dotted arrows in Figure 1 | | | |
